# Draft Whole-Genome Sequence of *Haemophilus ducreyi* Strain AUSPNG1, Isolated from a Cutaneous Ulcer of a Child from Papua New Guinea

**DOI:** 10.1128/genomeA.01661-15

**Published:** 2016-02-04

**Authors:** Dharanesh Gangaiah, Georgi K. Marinov, Sally A. Roberts, Jenny Robson, Stanley M. Spinola

**Affiliations:** a Department of Microbiology and Immunology, Indiana University School of Medicine, Indianapolis, Indiana, USA; b Department of Biology, Indiana University, Bloomington, Indiana, USA; c Department of Microbiology, Auckland District Health Board, Auckland, New Zealand; d Department of Microbiology, Sullivan Nicolaides Pathology, Brisbane, Queensland, Australia; e Department of Medicine, Indiana University School of Medicine, Indianapolis, Indiana, USA; f Department of Pathology and Laboratory Medicine, Indiana University School of Medicine, Indianapolis, Indiana, USA; g Center for Immunobiology, Indiana University School of Medicine, Indianapolis, Indiana, USA

## Abstract

*Haemophilus ducreyi* has recently emerged as a leading cause of cutaneous ulcers in the yaws-endemic areas of Papua New Guinea and other South Pacific islands. Here, we report the draft genome sequence of the *H. ducreyi* strain AUSPNG1, isolated from a cutaneous ulcer of a child from Papua New Guinea.

## GENOME ANNOUNCEMENT

*Haemophilus ducreyi* causes the genital ulcer disease chancroid and has emerged as a leading cause of cutaneous ulcers (CU) in the yaws-endemic regions of the South Pacific islands and equatorial Africa (1–3). By whole-genome sequence analysis, CU strains from Samoa and Vanuatu are almost identical to the genital ulcer (GU) strain 35000HP, and CU strains form a subcluster within the class I clade of GU *H. ducreyi* (4). This study was limited by the lack of CU strains from other countries. Here, we report the draft genome sequence of *H. ducreyi* AUSPNG1, which was isolated in 2013 from a CU of a 12-year-old boy from Papua New Guinea, who was treated in Brisbane, Australia.

AUSPNG1 was grown on GC medium base agar plates (Difco, Becton, Dickinson) supplemented with 1% bovine hemoglobin (Sigma-Aldrich) and 1% GCHI enrichment (Remel, Thermo Fisher) at 33°C with 5% CO_2_. Genomic DNA was extracted using the MagNApure nucleic acid extraction kit (Roche). The libraries were prepared using the NexteraXT DNA library preparation kit (Illumina) and sequenced on the MiSeq platform as paired 250-bp reads. Adapter sequences and low-quality bases were trimmed using Trimmomatic version 0.33 (5). Reads were error-corrected using BayesHammer and assembled using SPAdes version 3.5.0, Edena version 3.131028, and IDBA version 1.1.1; assemblies were then integrated using CISA version 1.3 (6–10). Protein-coding genes were annotated using RAST (11). rRNAs were annotated using RNAmmer version 1.2 and tRNAs using tRNAscan-SE version 1.3.1 (12, 13). Additional noncoding RNAs were annotated using Infernal version 1.1.1 and the Rfam database version 12.0 (14, 15). The contigs were ordered and aligned against the reference genome 35000HP using Mauve (16, 17). Phylogenetic analysis was performed by the maximum likelihood method using Mega version 6.0 (18).

After filtering out potential contaminants, the final assembly consisted of 26 contigs (the largest being 480,771 bp) with a coverage of ~294×, total length of 1,727,680 bp, GC content of 37.5%, *N*_50_ of 268,281 bp, and *N*_90_ of 35,093 bp. Annotation resulted in 1,697 protein-coding genes, 48 tRNAs, 7 5S rRNAs, 6 16S rRNAs, 6 23S rRNAs, and 31 other ncRNAs. Phylogenetically, AUSPNG1 belongs to the CU subcluster within the class I clade and is most related to 35000HP among the GU strains and to NZV1 among the CU strains. A circular plasmid with 99% nucleotide identity to pB1000 of *Haemophilus parasuis* and containing the β-lactam resistance gene *bla*ROB-1 was noted; β-lactamase production was confirmed using the BBL Cefinase paper discs (Becton, Dickinson). This is the first report of a CU strain expressing β-lactamase, which is disturbing in that penicillin is frequently used for empirical treatment of CU in the tropics (4, 19, 20).

The availability of the AUSPNG1 genome sequence will facilitate additional studies to better understand the epidemiology, pathogenesis, evolution, and prevention of *H. ducreyi*-associated CU in yaws-endemic regions.

### Nucleotide sequence accession numbers.

This whole-genome shotgun project has been deposited at DDBJ/EMBL/GenBank under the accession number LMZZ00000000. The version described in this paper is the first version, LMZZ01000000.
